# Primary Early‐Stage Basal Cell Carcinoma of the External Auditory Canal and the Role of Sleeve Resection: Case Series and Review of the Literature

**DOI:** 10.1155/crot/9932974

**Published:** 2026-10-20

**Authors:** Daniele Fasanella, Enrico Liaci, Maria Luce Bardon, Andrea Bacciu, Enrico Pasanisi, Filippo Di Lella

**Affiliations:** ^1^ University of Parma Parma Italy unipr.it

**Keywords:** audiometry, carcinoma basal cell, external auditory canal, surgical procedures operative, temporal bone

## Abstract

**Objectives:**

Primary basal cell carcinoma (BCC) of the external auditory canal (EAC) is an uncommon malignancy for which evidence‐based treatment recommendations remain limited. This study reports two institutional cases treated with sleeve resection and provides a structured review of the published literature, with particular emphasis on patient selection, surgical technique and the limitations of the currently available evidence.

**Methods:**

A retrospective review was performed of patients with primary BCC of the EAC treated at the Otolaryngology and Otoneurosurgery Department, Parma University Hospital (Italy). In addition, a structured review of the literature based on a systematic search of PubMed and Google Scholar was conducted. Only histologically confirmed primary BCCs arising from the EAC were included. Institutional and published cases were summarised descriptively with regard to tumour stage, treatment modality, recurrence, postoperative complications, adjuvant therapy and follow‐up.

**Results:**

Two patients with early‐stage primary BCC of the EAC underwent sleeve resection with intraoperative frozen‐section margin assessment and remained disease‐free after 24 and 36 months of follow‐up, respectively. One patient developed partial postoperative EAC stenosis. The literature search identified 11 additional patients from five published studies, resulting in a descriptive cohort of 13 cases. Conservative surgical approaches, including local canal resection and sleeve resection, were the most frequently reported treatments. Only one recurrence was identified across the available reports. However, treatment strategies and follow‐up duration were highly heterogeneous, precluding meaningful comparisons between conservative and more extensive surgical approaches.

**Conclusion:**

Sleeve resection appears to be a feasible hearing‐preserving option for carefully selected patients with early‐stage primary BCC confined to the EAC and without radiological or intraoperative evidence of bony invasion. However, the currently available evidence is limited to isolated case reports and small retrospective series. Consequently, no conclusions regarding oncological equivalence with more extensive surgery can be drawn. Careful preoperative staging, intraoperative margin assessment and long‐term clinical surveillance extending beyond 5 years remain essential.

## 1. Introduction

Carcinomas of the external auditory canal (EAC) are exceedingly rare, accounting for only a small fraction of head and neck malignancies [1]. Among these, primary basal cell carcinoma (BCC) of the EAC represents an exceptionally uncommon entity, with an estimated incidence of fewer than one case per million population per year [2]. Only a limited number of case reports and small retrospective series have described BCC arising primarily within the EAC, and standardised treatment recommendations remain unavailable. Consequently, management is largely based on institutional experience and extrapolation from other temporal bone malignancies. Treatment planning should aim to achieve complete oncological clearance while preserving hearing whenever oncologically appropriate.

BCC originates from the basal layer of the epidermis and is characterised by slow growth, local invasiveness and an overall low metastatic potential, particularly when diagnosed at an early stage [3]. Within the spectrum of temporal bone malignancies, BCC belongs to the heterogeneous group of nonsquamous tumours, which also includes adenoid cystic carcinoma and neoplasms arising from cutaneous adnexal structures [4]. Although generally considered an indolent tumour, involvement of the EAC may increase the risk of local recurrence because of the anatomical proximity to the temporal bone and adjacent critical structures.

It is important to distinguish primary BCC of the EAC from secondary involvement of the canal by cutaneous BCC arising from the auricle, conchal bowl or adjacent periauricular skin. The latter represents a different clinical entity, with distinct patterns of local spread, surgical management and prognosis [5]. Throughout the present study, only histologically confirmed lesions with a clinical epicentre originated within the EAC were considered primary tumours. Cases with direct extension from neighbouring cutaneous sites or with uncertain tumour origin were excluded from the literature review.

Clinically, primary BCC of the EAC often presents with nonspecific symptoms, including otorrhoea, hypoacusis, aural fullness, pruritus, intermittent bleeding or otalgia, frequently resembling chronic inflammatory conditions such as otitis externa [6]. This nonspecific presentation may contribute to delayed diagnosis and highlights the importance of maintaining a high index of suspicion when persistent unilateral EAC lesions fail to respond to conventional medical treatment.

Lateral temporal bone resection (LTBR) has traditionally been regarded as the reference surgical treatment for malignant tumours of the EAC, particularly when bone invasion is suspected or confirmed. Although LTBR provides wide oncological margins, it is associated with considerable morbidity, including permanent conductive hearing loss due to sacrifice of the EAC and middle ear structures. Conversely, in carefully selected patients with early‐stage disease confined to the soft tissues of the EAC, more conservative procedures such as sleeve resection have been described as potential hearing‐preserving alternatives. However, the available evidence remains limited to isolated case reports and small retrospective series, precluding robust comparisons between different surgical strategies.

The aim of the present study was to report our institutional experience with sleeve resection in two patients with early‐stage primary BCC of the EAC and to provide a structured review of the available literature. Particular emphasis was placed on the technical aspects of sleeve resection and on the clinical features that may guide surgical decision‐making in this exceptionally rare disease.

### 1.1. Case Series

The present case series includes two patients affected by primary early‐stage BCC of the EAC who underwent sleeve resection at the Otolaryngology and Otoneurosurgery Department, Parma University Hospital, a tertiary referral centre for otologic surgery, between January 2015 and June 2024. Demographic characteristics, presenting symptoms, radiological findings, surgical management, histopathological results and clinical outcomes were retrospectively reviewed.

#### 1.1.1. Patient 1

A 64‐year‐old woman presented with a one‐year history of left‐sided hypoacusis, intermittent otorrhoea, and otorrhagia. Otoscopic examination identified a subocclusive polypoid lesion arising from the roof of the lateral third of the left EAC. Incisional biopsy established a diagnosis of BCC, and high‐resolution computed tomography (CT) and magnetic resonance imaging (MRI) demonstrated a lesion confined to the soft tissues of the lateral EAC, without evidence of bony erosion or extension to adjacent structures, consistent with T1 disease according to the modified Pittsburgh staging system. The patient underwent sleeve resection of the EAC with bony canaloplasty under general anaesthesia with facial nerve monitoring. Frozen‐section analysis confirmed negative proximal, distal and deep margins before reconstruction. The tumour was removed en bloc with macroscopically free margins (shown in Figure 1). The postoperative period was uneventful, and no signs of recurrence have been observed after 36 months of follow‐up.

**FIGURE 1:**
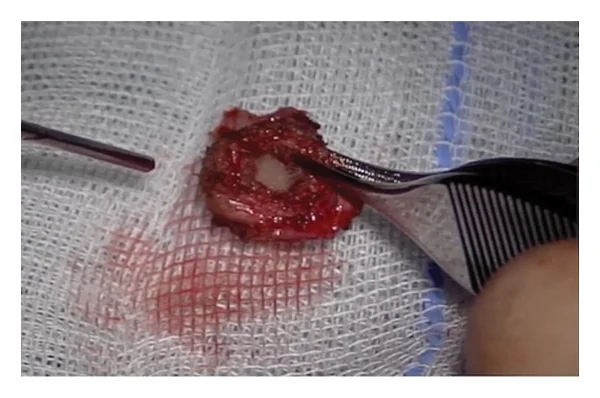
Patient 1—intraoperative picture. En bloc excision of the left EAC.

#### 1.1.2. Patient 2

A 77‐year‐old woman presented with left aural fullness and itching without hypoacusis or otalgia associated with a circumferential lesion of the EAC with a villous appearance. Incisional biopsy established a diagnosis of BCC, and imaging excluded lymph node and bone involvement. The lesion fulfilled the same selection criteria adopted for conservative management in Patient 1, and sleeve resection was therefore considered oncologically appropriate. A sleeve resection with canaloplasty was performed with no immediate postoperative complications. At the 3‐month postoperative visit, partial stenosis of the lateral EAC was observed. The patient remained asymptomatic, with preserved tympanic membrane integrity and no evidence of disease recurrence after 24 months of follow‐up (as shown in Figure 2a,b). In both patients, intraoperative frozen‐section analysis confirmed negative proximal, distal and deep margins. Final histopathological examination confirmed complete tumour excision with clear surgical margins, and no adjuvant treatment was indicated.

**FIGURE 2:**
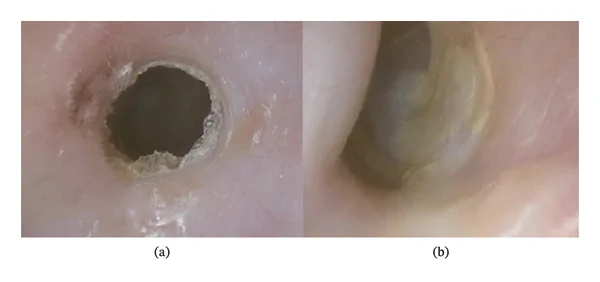
Patient 2—postoperative picture after sleeve resection of the left ear. Although partial EAC stenosis is a recognised postoperative complication of sleeve resection (a), the tympanic membrane was left intact (b), and postoperative hearing was preserved.

### 1.2. Patient Selection for Sleeve Resection

Sleeve resection was considered only when all the following criteria were fulfilled:

• histologically confirmed primary BCC arising within the EAC;
• lesion confined to the soft tissues of the EAC;
• absence of radiological evidence of bony erosion;
• absence of middle ear, mastoid, temporomandibular joint, or parotid involvement;
• feasibility of circumferential en bloc resection with intraoperative confirmation of negative margins.

### 1.3. Literature Search and Study Selection

The literature review protocol was not prospectively registered. According to the local institutional review board, formal ethical approval was not required for this retrospective investigation; however, a waiver was obtained (protocol no. 0020215, 5 May 2025). Written informed consent was obtained from both patients included in the study. The investigation was conducted in accordance with the Declaration of Helsinki and its subsequent amendments.

A structured review of the literature was performed following a systematic search strategy based on the Preferred Reporting Items for Systematic Reviews and Meta‐Analyses (PRISMA) 2020 recommendations. Given the rarity of the disease and the predominance of isolated case reports and small retrospective series, the objective of the review was to provide a descriptive synthesis of the available evidence rather than to compare treatment effectiveness. The literature search was performed on 18 June 2025, using PubMed and Google Scholar. The search strategy combined the following terms: (“basal cell carcinoma” OR “basalioma”) AND (“external auditory canal” OR “ear canal” OR “temporal bone”). Only articles published in English between January 2000 and December 2024 involving human subjects were considered. Studies were considered eligible when they fulfilled all of the following criteria:

1. histologically confirmed primary BCC originating from the EAC;
2. sufficient clinical information regarding tumour stage, treatment, and follow‐up;
3. clear distinction between primary EAC BCC and secondary extension from neighbouring cutaneous sites.

Data extraction included patient demographics, tumour stage according to the modified Pittsburgh staging system whenever available, surgical treatment, adjuvant therapy, recurrence, postoperative complications and duration of follow‐up.

Cases with uncertain tumour origin, involvement of the auricle as the primary site or insufficient clinical information were excluded from the descriptive synthesis.

Because the review focused on detailed clinicopathological characterisation and surgical outcomes, reports lacking essential clinical information were excluded. Nevertheless, exclusion of incomplete reports may have introduced selection bias and may have favoured publication of cases with more favourable outcomes. Owing to the rarity of the disease and the predominance of single‐case reports, a formal risk‐of‐bias assessment was not considered appropriate. Instead, the completeness of clinical reporting was qualitatively evaluated. The principal sources of bias were considered to include publication bias, incomplete reporting, retrospective data collection and selection bias related to the exclusion of incompletely documented cases.

The literature search and study‐selection process is illustrated in the PRISMA flow diagram.

Because of the limited number of available cases, heterogeneous reporting and absence of comparative studies, the literature review was intended to provide a descriptive overview of current evidence rather than a quantitative assessment of treatment efficacy.

## 2. Results

Over the period January 2004–June 2024, a total of 11 patients affected by primary BCC of the EAC from 5 studies met the inclusion criteria after detailed review by the authors from over 160 articles initially identified [2–6]. Several studies lacking the data mentioned above were excluded from the analysis, as shown in Figure 3.

**FIGURE 3:**
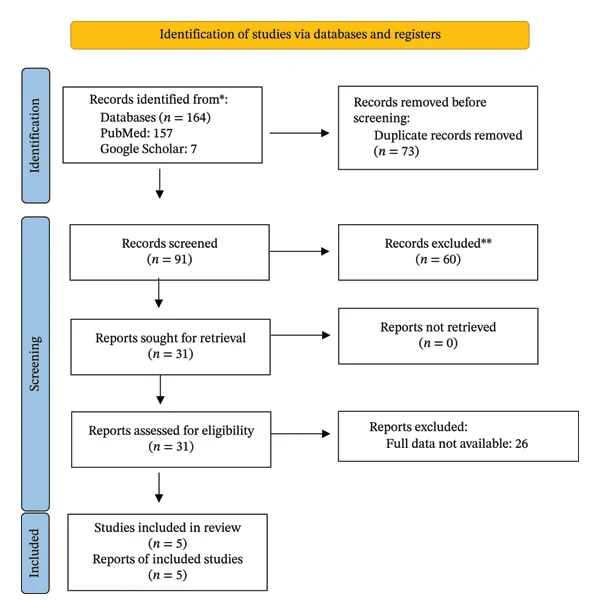
PRISMA diagram showing the process of the study selection.

### 2.1. Descriptive Analysis

The present descriptive analysis combined the two institutional cases with the cases identified through the literature search in order to summarise the currently available evidence regarding the management of primary early‐stage BCC of the EAC. Overall, 13 patients with histologically confirmed T1 disease according to the modified Pittsburgh staging system were included (Table 1). The mean age was 69.5 years (range 51–84 years), and female patients accounted for 77% of the cohort. Conservative surgery was the most frequently reported treatment. Local canal resection was performed in seven patients (53.8%), while formal sleeve resection was adopted in three patients (23.1%), including the two cases presented in the current series. More extensive surgical procedures consisted of one total canal resection and one subtotal petrosectomy. One patient underwent photodynamic therapy. One case of partial EAC stenosis was reported as a postoperative complication. Follow‐up duration was markedly heterogeneous, ranging from 4 to 80 months. One recurrence was reported 55 months after treatment in the patient who underwent subtotal petrosectomy. No patient received adjuvant radiotherapy.

**TABLE 1:** Summarised pooled data of cases extrapolated from scientific literature.

Author	Year	N° patients	Age	Sex	Treatment	Adjuvant RT	Recurrence	Complications	Staging	Follow‐up (months)
Park et al.	2018	1	61	F	LCR	—	—	—	T1	80
Park et al.	2018	1	78	F	LCR	—	—	—	T1	4
Park et al.	2018	1	84	F	LCR	—	—	—	T1	5
Park et al.	2018	1	73	F	LCR	—	—	—	T1	8
Park et al.	2018	1	61	M	LCR	—	—	—	T1	22
Park et al.	2018	1	51	M	LCR	—	—	—	T1	9
Park et al.	2018	1	70	F	LCR	—	—	—	T1	8
Lee et al.	2012	1	55	F	SR	—	—	—	T1	12
Hosokawa et al.	2012	1	73	F	TCR	—	—	—	T1	78.3 m
Correia et al.	2020	1	78	M	Ptr	—	After 55 months	2nd surgery	T1	55
Xu et al.	2019	1	80	F	PhT	—	—	—	T1	12
Present series	2026	1	77	F	SR	—	—	Partial EAC stenosis	T1	24
Present series	2026	1	64	F	SR	—	—	—	T1	36

*Note:* M, male; F, female; Ptr, petrosectomy; PhT, photodynamic therapy; m, median.

Abbreviations: LCR, local canal resection; SR, sleeve resection.

Because of the small sample size, heterogeneous treatments, unequal follow‐up duration and the occurrence of only one recurrence event, no inferential statistical comparisons between treatment groups were considered appropriate. Overall, the available evidence suggests that conservative surgery may be feasible in carefully selected early‐stage lesions; however, the current literature remains insufficient to establish comparative oncological outcomes between conservative and more extensive surgical approaches.

### 2.2. Sleeve Resection: Surgical Technique

The detailed step‐by‐step description of sleeve resection, together with the intraoperative photographs provided in the present report, may facilitate reproducibility of this technique in centres managing selected temporal bone malignancies.

Sleeve resection may be considered for selected early‐stage (T1) lesions confined to the EAC without bony or middle‐ear involvement. Its main advantage is hearing preservation. The procedure is performed through a retroauricular approach: The lateral third of the EAC is circumferentially incised, and the skin with tumour is removed en bloc, keeping a 1–2‐mm margin from the tympanic membrane. Intraoperative frozen sections are routinely obtained to confirm negative margins. A canaloplasty of the tympanic bone is then performed to eliminate microscopic disease and reduce recurrence risk. Reconstruction involves covering the exposed bone with temporalis fascia, placing a retroauricular skin graft and inserting silastic sheets to support healing. Finally, a wide conchal meatoplasty is performed to prevent postoperative stenosis (Figure 4a–d).

**FIGURE 4:**
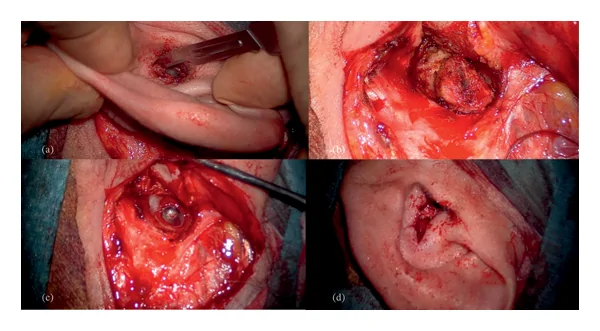
Patient 1—steps of sleeve resection: lateral incisional border (a); opened external auditory canal, with circumferential lesion surrounding the bony external auditory canal visible (b); external auditory canal after sleeve resection and canaloplasty (c); and meatoplasty (d).

Although no evidence‐based indications can currently be established, our experience, together with the available literature, suggests that sleeve resection may be considered in highly selected patients fulfilling a number of clinical and radiological criteria. The proposed selection criteria shown in Table 2 are intended as a practical aid for surgical decision‐making rather than as formal treatment recommendations.

**TABLE 2:** Proposed institutional criteria to guide consideration of sleeve resection for early‐stage primary BCC of the EAC.

Criterion	Rationale
Histologically confirmed primary BCC	Exclude secondary skin extension
T1 according to modified Pittsburgh	Limited disease
No bone erosion on CT	Conservative surgery feasible
No middle ear involvement	Hearing preservation
Negative frozen sections achievable	Oncological safety
Long‐term follow‐up planned	Late recurrence possible

## 3. Discussion

Primary BCC of the EAC is an exceptionally rare malignancy; consequently, the available evidence is limited to isolated case reports and small retrospective series. In this context, the present study combines two additional institutional cases with a structured review of the literature, providing an updated descriptive overview of the clinical presentation, surgical management and outcomes of early‐stage disease.

Radical surgical excision remains the gold standard for BCC of the EAC [7]. Adjuvant radiotherapy and, more rarely, chemotherapy are reserved for advanced disease, particularly in cases with positive margins, unresectable tumours or as palliation [8]. The University of Pittsburgh modified staging system is the most widely used classification, guiding both surgical management and prognosis [4]. Evidence specific to primary BCC of the EAC is scarce, and standardised protocols are lacking. LTBR is traditionally considered the preferred approach for malignancies extending beyond soft tissues [9]. However, in early‐stage disease, conservative options such as local canal resection or sleeve resection have been reported [2, 10]. Breen et al. described three local recurrences managed successfully with limited surgical margin enlargements, without recourse to demolitive surgery [10]. Although sleeve resection has been associated with favourable outcomes in selected patients, the currently available evidence is insufficient to establish oncological equivalence with more extensive procedures such as LTBR. The rarity of primary EAC BCC and the predominance of isolated case reports preclude robust comparative analyses. Sleeve resection allows hearing preservation but carries risks such as meatal stenosis, preventable by adequate meatoplasty. Reconstruction with free skin grafts or temporalis fascia may predispose to cholesteatoma if margins are not properly managed. Because sleeve resection entails a more limited surgical field than LTBR, meticulous intraoperative margin assessment and prolonged postoperative surveillance are particularly important. Interestingly, the only recurrence identified in the pooled cohort occurred after 55 months of follow‐up, suggesting that shorter surveillance periods may underestimate the true recurrence rate. Consequently, prolonged postoperative follow‐up extending beyond 5 years appears advisable irrespective of the surgical approach adopted. The ‘Data Set for the Reporting of Ear and Temporal Bone Tumours’ endorses sleeve resection for early lesions confined lateral to the tympanic membrane without bony involvement [11]. In our descriptive synthesis, favourable outcomes were reported among selected early‐stage tumours treated conservatively; however, these observations should be interpreted cautiously given the small number of cases, heterogeneous follow‐up and potential publication bias. In the cohort examined, only one partial stenosis occurred, and no adjuvant therapy was required. Rather than demonstrating superiority of one surgical strategy over another, the present study provides practical information regarding patient selection and surgical decision‐making. In our experience, sleeve resection should only be considered in patients with disease confined to the soft tissues of the EAC, without radiological evidence of bony erosion and when complete circumferential excision with negative frozen‐section margins can reasonably be achieved.

## 4. Limitations

Several limitations should be acknowledged:

1. Primary BCC of the EAC is an exceptionally rare disease, resulting in a very limited number of available cases.
2. The evidence derives almost exclusively from retrospective case reports and small case series, with heterogeneous follow‐up duration and outcome reporting.
3. Exclusion of incompletely documented reports may have introduced selection bias and favoured publication of cases with more favourable outcomes.
4. The descriptive nature of the available evidence precludes meaningful comparison between different surgical strategies.

## 5. Conclusions

This report adds two further cases of primary early‐stage BCC of the EAC successfully managed with sleeve resection and contributes a structured synthesis of the currently available literature. Sleeve resection may represent a feasible hearing‐preserving option in carefully selected patients with disease confined to the EAC and without radiological evidence of bony involvement. However, the available evidence remains limited and descriptive, preventing conclusions regarding comparative oncological effectiveness. Careful patient selection, meticulous intraoperative margin assessment and long‐term postoperative surveillance extending beyond 5 years remain essential for optimal clinical management.

## Funding

The authors received no financial support for the research, authorship and/or publication of this article.

## Conflicts of Interest

The authors declare no conflicts of interest.

## Data Availability

The data that support the findings of this study are available from the corresponding author upon reasonable request.
